# Current Hospital Topics

**Published:** 1906-05-26

**Authors:** 


### CURRENT HOSPITAL TOPICS.

### A Diagram of Authority.

The management of hospitals is now exciting a great deal of attention in the United States of America. Dr. Ochsner, a hospital surgeon of Chicago, has written several papers on the subject in recent years, and in his latest publication just issued he insists upon the importance of treating hospitals as business enterprises, and securing that all persons performing work in the hospital shall be properly paid for their services. He maintains that adequate payment is essential in order to make it proper to expect good work from every one employed, and to secure that every one who fails to do his share of it shall be dismissed in justice to the worker and for the benefit of the institution. The smooth working of a great establishment like that of a large hospital depends largely upon each employé understanding exactly how the organisation is built up, and in whose department each item of the work ought to be undertaken. To secure this our American cousins have evolved a diagram of authority, which shows at a glance each person connected with the establishment under whose direct supervision the duties are to be performed, and who in turn is expected to work under the supervision of each responsible official. This diagram of authority establishes diagramatically who is the superior in command throughout the system. The great advantage of such a scheme is apparent. Every employé knows at a glance to whom he must apply for advice and orders, and to whom in turn his orders must be given, the name in each instance being opposite the position occupied by each individual employé. Where this plan is followed there can be no conflict between authorities, because each person must know at once what his exact position is. Dr. Ochsner publishes diagrams of authority for large and small hospitals. At the head is the board of directors, and immediately under them are the three great departments, vested in the secretary, the president and the treasurer. The duties of the first and last are identical with those assigned to similar officers in this country. The administration of the whole of the internal departments of the hospital is vested in the president, with powers identical with those given to the treasurer of an endowed hospital in England. Under the direction of the president the medical superintendent takes charge of the medical arrangements of the nursing department with the assistance of a superintendent of nurses, of the resident staff including interns and externs, and of the heads of the various medical departments. Next come the various committees "according to requirements—the fewer the better"; then the superintendent of the hospital, who is responsible for the commissariat, the structural and engineering departments, and the extension and maintenance of the fabric. We think this suggestion of a diagram of authority to be suspended in various parts of the hospital a good one, and we have little doubt that it will commend itself to the judgment of those who study the plan and make themselves familiar with its working.

### Newspapers and the Hospitals.

No doubt the increase in the number of newspapers and the advent of halfpenny journals may be held accountable for much good, and also for many things which are neither good nor desirable. It cannot be too often insisted upon that a newspaper usually is a business venture, and those who invest their money in it look for a good and increasing profit. The larger the circulation the greater the return on the money invested, a fact which has led in some countries, and has tended in this country, to weaken the influence of newspapers by causing sensation and sentiment to dominate more and more the matter published, to the exclusion of sobriety and facts. Some daily journals indeed seem to consider it smart to publish anything of a sensational character about anybody or any institution without waiting to test its accuracy on the ground that any mis-statement can be corrected on the morrow or elaborated on succeeding days whereby interesting copy is readily manufactured. Unfortunately too newspaper readers consist in great number of individuals who seem to accept everything which appears in print as gospel, and to circulate it without thought or hesitation. So it has happened that the hospitals have suffered very often from the publication of unwarrantable statements, and by the circulation of figures which will not bear the test of examination. There is no more favourite fancy nowadays than to represent the hospitals of London, for instance, as languishing for want of funds, whereas in fact with very few exceptions there never was a time in their history when the public gave more largely to this class of institution, or when as a whole their finances were in a sounder condition than they are to-day. This is shown from the fact that, whereas the expenditure of 94 voluntary hospitals in the county of London increased from £837,725 in 1894 to £1,341,641 in 1903, the total available income increased from £807,785 in 1894 to £1,331,090 in 1903.

"Truth" on Hospital Finance.

*Truth*, referring to an article in the *Daily Mail*, asks whether the stream of public charity is drying up so far as hospitals are concerned? *Truth* proceeds to state that many of the hospitals are in serious financial straits, but this view is not supported by the ten years' figures of income and expenditure which will be found in Chapter I. of Burdett's "Hospitals and Charities" for 1905, and in the complementary Chapter I. of the volume of this book recently issued for 1906. These figures show that the ordinary income of the 94 hospitals in the county of London has increased from £608,362 in 1896, the year before the King's Fund was founded, to £741,557 in 1903. When the extraordinary income was taken into account, including legacies, it appears that the income from this source, with one exception, the year 1896, has been greater each year than the year 1894, rising from £133,500 in the latter year to £283,000 in 1903. The income of the 94 hospitals, including legacies, therefore increased from £752,942 in 1894 to £1,025,051 in 1903, an increase of £272,000 in ten years. Besides this a sum of £306,039 was collected for special purposes in 1903 as compared with only £54,843 in 1894, so that the total money contributed to the London hospitals in 1903 was £1,331,090 as compared with £807,785 in 1894, an increase of nearly £525,000, or 65 per cent. more in 1903 than in 1894. The total expenditure of these 94 hospitals in the county of London has, however, increased from £837,725 in 1894 to £1,341,641 in 1903. *Truth* further suspects that the starting of the King's Fund and the increase in the Hospital Sunday collections have tended to draw away subscriptions from individual hospitals. This view is shown to be without foundation by the figures contained in Burdett's "Hospitals and Charities" already referred to. The figures confirm the view that there is little if any real justification for insisting that the amount of money in any particular field of charity in the course of one year is limited, nor for the conclusion that pre-existing subscriptions have been diverted from individual hospitals to the general funds. The latter two assertions are stock arguments with certain critics who do not seem capable of taking the trouble to make themselves familiar with the actual facts governing the financial position of the voluntary hospitals of London at the present time.

